# Supplementary material for: Causal Relationship between Aging and Anorexia Nervosa: A White-Matter-Microstructure-Mediated Mendelian Randomization Analysis
Source: Biomedicines. 2024 Aug 16;12(8):1874. doi: 10.3390/biomedicines12081874 (PMC11351342; doi:10.3390/biomedicines12081874)
Supplement: Supplementary file 1 [file biomedicines-12-01874-s001.zip › Supplementary Figure/Figure S4/Singe SNP plot of MR analysis for TL to ubm-b-1810.pdf]

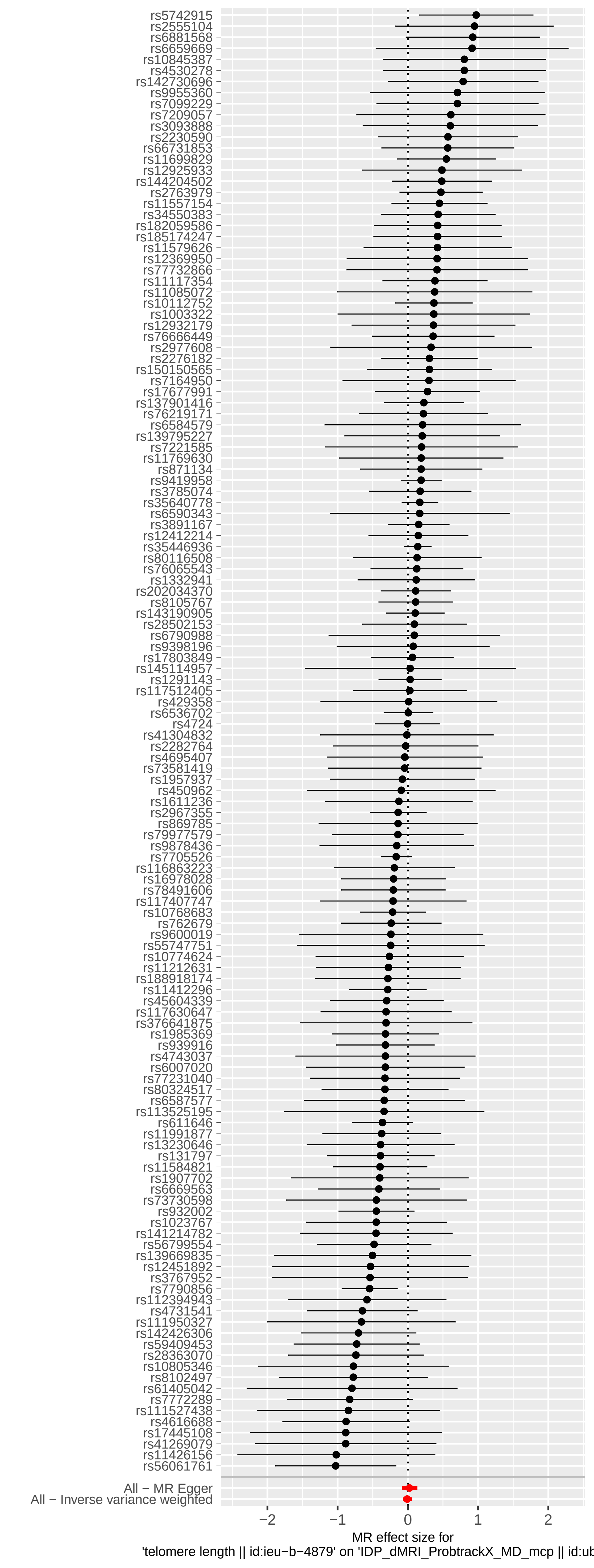
rs5742915
rs2555104
rs6881568
rs6659669
rs10845387
rs4530278
rs142730696
rs9955360
rs7099229
rs7209057
rs3093888
rs2230590
rs66731853
rs11699829
rs12925933
rs144204502
rs2763979
rs11557154
rs34550383
rs182059586
rs185174247
rs11579626
rs12369950
rs77732866
rs11117354
rs11085072
rs10112752
rs1003322
rs12932179
rs76666449
rs2977608
rs2276182
rs150150565
rs7164950
rs17677991
rs137901416
rs76219171
rs6584579
rs139795227
rs7221585
rs11769630
rs871134
rs9419958
rs3785074
rs35640778
rs6590343
rs3891167
rs12412214
rs35446936
rs80116508
rs76065543
rs1332941
rs202034370
rs8105767
rs143190905
rs28502153
rs6790988
rs9398196
rs17803849
rs145114957
rs1291143
rs117512405
rs429358
rs6536702
rs4724
rs41304832
rs2282764
rs4695407
rs73581419
rs1957937
rs450962
rs1611236
rs2967355
rs869785
rs79977579
rs9878436
rs7705526
rs116863223
rs16978028
rs78491606
rs117407747
rs10768683
rs762679
rs9600019
rs55747751
rs10774624
rs11212631
rs188918174
rs11412296
rs45604339
rs117630647
rs376641875
rs1985369
rs939916
rs4743037
rs6007020
rs77231040
rs80324517
rs6587577
rs113525195
rs611646
rs11991877
rs13230646
rs131797
rs11584821
rs1907702
rs6669563
rs73730598
rs932002
rs1023767
rs141214782
rs56799554
rs139669835
rs12451892
rs3767952
rs7790856
rs112394943
rs4731541
rs111950327
rs142426306
rs59409453
rs28363070
rs10805346
rs8102497
rs61405042
rs7772289
rs111527438
rs4616688
rs17445108
rs41269079
rs11426156
rs56061761
All − MR Egger
All − Inverse variance weighted
−2
−1
0
1
2
MR effect size for
'telomere length || id:ieu−b−4879' on 'IDP_dMRI_ProbtrackX_MD_mcp || id:ub
